# Differences in polysomnographic, nocturnal penile tumescence and penile doppler ultrasound findings in men with stuttering priapism and sleep-related painful erections

**DOI:** 10.1038/s41443-021-00462-3

**Published:** 2021-08-13

**Authors:** Mark Johnson, Venkata McNeillis, Julia Gutbier, Andy Eaton, Robert Royston, Thomas Johnson, Giovanni Chiriaco, Miles Walkden, David Ralph

**Affiliations:** University College London Hospitals, London, UK

**Keywords:** Sexual dysfunction, Reproductive signs and symptoms

## Abstract

Men with Stuttering Priapism (SP) and sleep-related painful erections (SRPE) experience bothersome nocturnal painful erections resulting in poor sleep. The aim of this study is to observe common features and differences between men with SP and SRPE based on polysomnography, nocturnal penile tumescence (NPT), and penile doppler ultrasound (PDU). This is a prospective cohort study of 20 participants divided into two groups (Group 1 = SP [*n* = 12]; Group 2 = SRPE [*n* = 8]) with bothersome painful nocturnal erections. All participants were referred to the sleep disorder clinic to be assessed and consented for overnight polysomnography with simultaneous NPT recording and to complete validated sleep, sexual dysfunction and health-related quality of life questionnaires. Unstimulated PDU was also performed. Abnormal Polysomnographic findings (reduced sleep efficiency, total sleep time, and awake after sleep onset) were identified in both groups suggesting poor sleep. Men with SP had significantly longer erections (60.0 vs 18.5; *p* = 0.002) and took longer to detumesce once awake (25.7 vs 5.4 min; *p* = 0.001) than men with SRPE. They also had significantly higher peak systolic and end diastolic velocities on unstimulated PDU with an abnormal low resistance waveform identified. No sleep pathology was identified in men with SP. This implies a local (penile) etiology in men with SP. Men with SRPE had a normal resting PDU and abnormal sleep architecture with REM awakenings and significantly more Periodic limb movements (*p* = 0.04) than men with SP suggesting a central (sleep-related) cause in men with SRPE. Sexual dysfunction and poor HR-QoL was identified on validated questionnaires in both groups. SP and SRPE are rare entities that share similar symptoms (painful nocturnal erections and poor sleep) but dissimilar features of nocturnal erection onset, duration and resolution with different polysomnographic features which may allude to a different pathophysiology.

## Introduction

A chief complaint of men with both stuttering priapism (SP) and sleep-related painful erections (SRPE) is poor sleep and daytime tiredness. Poor sleep is associated with increased cardiovascular disease, stroke, cancer, mental health problems, dementia, sepsis, and all-cause mortality [1, 2].

SP is a subtype of ischemic priapism (IP) that is typified by recurrent, self-limiting, painful penile erections that often require manoeuvres (exercise, voiding, or cold shower) to aid detumescence. It is accepted that episodes that progress beyond 4 h require emergency decompression in line with the current guidelines for management of IP. These recurrent episodes can occur at any time however typically occur during sleep, with the erection causing ischemic pain and waking the patient [3]. SPRE is an idiopathic condition characterized by recurrent and painful sleep related erections. These typically occur during REM sleep and differ from the painless erections these men experience when awake. Men with SRPE do not experience episodes of IP [4, 5].

It can be difficult to differentiate between these two conditions due to the similarities in their clinical presentation and definitions as highlighted above. This is further exacerbated by the rarity of these conditions, multidisciplinary involvement (Urologists, Hematologists, and Sleep physicians) and limited experience outside of expert centers [6, 7]. These factors combined can make accurate diagnosis difficult and can result in over- or under-treatment if managed incorrectly.

The goal in the management of men with both conditions is symptomatic relief by reducing the frequency and duration of episodes; improving the pain experienced and improving the patients sleep. Prevention of episodes of prolonged IP with the associated morbidity is a further key goal on the management of men with SP.

This is a hypothesis-generating observational study to describe the similarities and differences between men with SP and SRPE based on polysomnography, nocturnal penile tumescence (NPT) testing, Penile Doppler Ultrasound (PDU), and validated health-related quality of life questionnaires.

## Patients and methods

A prospective cohort study of 20 men with uncontrolled painful nocturnal erections. Participants were recruited over a 2-year period (2016–8) into two groups: SP group (*n* = 12) contains men that have previously experienced episodes of IP (>4 h) confirmed with aspiration of the corpus cavernosa. SRPE group (*n* = 8) contains men that report bothersome painful nocturnal erections, but have never experienced an episode of IP or had any emergency priapism intervention. Patients that were <18 years old were excluded from the study.

All had a clinical assessment performed at a tertiary andrology center. Their symptoms were treated in accordance with usual practice and guidelines [3]. The study inclusion criteria were; men > 18 years old, recurrent episodes of painful nocturnal erection (≥1 episode per week) and sleep disturbance. Men with sleep disturbance were identified if they scored ≥ 5 on the Pittsburgh Sleep Quality Index (PSQI) [8] and were referred onward to our institution’s sleep unit for assessment of their sleep history and simultaneous polysomnography and NPT.

All participants in this study were seen by an experienced sleep physician and verbally consented in clinic to undertake the polysomnography with NPT. Each participant also completed an International Index of Erectile Function [9] [IIEF] and Short-form 36 Health Survey [SF36] [10] validated questionnaire just prior to their sleep study.

This study is registered with the local clinical governance and audit lead. Following discussion with the local research and ethics department, formal ethical approval was deemed not required. The tertiary service opted to change its practice to undertake simultaneous polysomnography and NPT on the basis of strong evidence from the literature [7, 11–17].

### Polysomnography

All participants in this study underwent simultaneous polysomnography and NPT recording as an inpatient. They were given a quiet side room and were advised to avoid alcohol and caffeine prior to attendance. Analysis and scoring of the polysomnography was performed by an independent clinical scientist and verified by a Consultant Sleep Physician.

A Noxturnal (NOX, USA) A1 portable polysomnography system was used to record electroencephalograms (EEG) (C3-A2, C4-A1, and O1-A2 were used for analysis); submental and bilateral tibial electromyograms (EMG); and bilateral electro-oculo-grams (EOG). This has ten channels of unipolar EEG/EOG and three unipolar EMG with four bipolar inputs with a sampling rate of 256 Hz. The polysomnography criteria met American Association of Sleep Medicine standards for extended polysomnography recording [8]. Nasal air flow, chest and abdominal movement and electrocardiogram (ECG) was also measured and recorded. Peripheral pulse oximetry was used to measure the patients’ oxygen saturations and a nasal cannula was used to measure nasal air flow. Measured polysomnographic parameters included sleep stage (REM, S1, S2 and S3); Total sleep time (TST); Total recording time (TRT); Sleep efficiency (TST/TRT × 100), Wake after sleep onset; sleep and REM latencies. Arousals were measured and categorized into respiratory and periodic limb movements (PLM). Scoring of an arousal required an increased frequency of EEG for ≥3 s with a minimum of 10 s preceding stable sleep. PLM of >15 arousals per hour is deemed abnormal. The following respiratory indices were recorded; Respiratory rate, oxygen saturation, apnoeas, hypopneas, apnoeas and hypopnea index (AHI) (number of events per hour). An AHI of >5 events per hour is considered abnormal and is diagnostic for obstructive sleep apnoea (OSA). Cardiovascular parameters were measured from ECG and plethysomography.

### Nocturnal penile tumescence

NPT (RigiScan^™^, USA) was used to measure the rigidity of the penile shaft. Two rings that intermittently constrict were each placed at the base of the penis and sub-coronal area. Intermittent constriction of the rings measured the radial rigidity (0–100%). Radial rigidity is a proxy of intracavernosal vascular resistance [18]. The time of onset, duration, and rigidity of each penile event was recorded and the data were correlated with the polysomnography findings at that time of each event.

### Penile doppler ultrasound

PDU studies were performed using a 12–18 MHz linear probe on an Accuson 500 (Siemens, Germany) machine. All of the studies were performed by specialist Uro-radiologists. The Doppler waveforms of the cavernosal arteries were measured bilaterally within the paired corpus cavernosa, close to their entry point at the crura over a single cardiac cycle. Angle correction was applied and a trace of the velocity measured for each side [19]. The velocities and shape of the waveform reflects the peripheral vascular resistance and tone. The peak systolic velocity (PSV) and end diastolic velocity (EDV) were recorded for each cavernosal artery. Given the risk of iatrogenic IP, all PDU were unstimulated. Men that had a PDU performed acutely (during an episode of IP) were excluded. In total 18 men (36 cavernosal arteries) underwent a PDU during the study period. The PDU studies were performed sporadically during the patient’s follow-up.

### Statistical analysis

All analyses were completed using R 3.2.3 with the package Stats 3.2. Graphs were produced using ggplot 2.0.0. When the data were evenly distributed, mean ± standard deviation and *T*-Test were used. When the data were not evenly distributed, median (Interquartile range) and Mann and Witney U-Test were used unless otherwise stated.

## Results

There was no significant difference between the two groups in terms of age at recruitment, age at diagnosis, and body mass index. For baseline demographics of the patients, see Table 1.

**Table 1:** Baseline demographic information.

Groups		Stuttering Priapism (*n* = 12) median ± IQR	SRPE (*n* = 8) median ± IQR
Age (years)^a^	39 ± 9		43 ± 21
Age at Diagnosis (years)^a^	28 ± 11		34 ± 21
Body Mass Index^a^	23.8 ± 5.7		23.9 ± 2.3
Follow up (months)	61 ± 13		47 ± 21
Comorbidities	SCD (*n* = 5) Schizophrenia (*n* = 1) Hypertension (*n* = 1)		SCD (*n* = 1) Depression/Anxiety (*n* = 3) GORD (*n* = 2)
Ethnicity	African/Caribbean (*n* = 5). White European (*n* = 5). South Asian (*n* = 2)		African/Caribbean (*n* = 3) White European (*n* = 4) South Asian (*n* = 1)

*SRPE* Sleep related painful erections, *IQR* Interquartile range, *SCD* Sickle cell disease, *GORD* Gastro-oesophageal reflux disease.

^a^*p* > 0.05 (Mann and Witney U-Test).

### Duration of erection

**Fig. 1:**
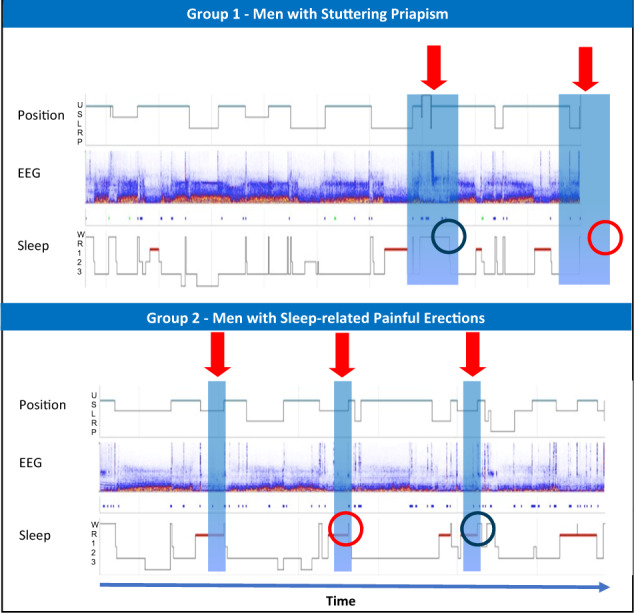
Differences in sleep architecture and duration/timings of erection measured using polysomnography and Nocturnal Penile Tumescence in men with Stuttering Priapism and Sleep related painful erections. Position (Up = upright; S = Supine; L = Left; R = Right; P = Prone), Sleep Stage (W = awake; R = REM; 1 = Stage 1; 2 = Stage 2; 3 = Stage 3). Red horizontal lines = REM sleep. Blue bars = Penile erections as determined from NPT: Longer duration of erections in Group 1 compared to Group 2. Red Circle = Awakenings during REM in Group 2; No awakenings during REM in Group 1. Blue Circle—Duration of erection after waking up; Longer in Group 1 compared to Group 2. Red arrows—Erectile events in second half of nights sleep in Group 1 and spaced out more evenly in Group 2.

Based on NPT findings, men with SP had a significantly longer total duration of nocturnal erection than men with SRPE (60.0 min vs 18.5 min; *p* = 0.002). Furthermore, once awake, men in the SP group took a significantly longer time to detumescence than men in the SRPE Group (25.7 vs 5.4 min; *p* = 0.001). In addition, men with SP tended to experience painful erections in the second half of their nights’ sleep whereas, the painful erectile events in men with SRPE tended to be more evenly spaced throughout the night (see Fig. 1). This was assessed qualitatively.

### Polysomnographic findings

The salient sleep parameters are listed in Table 2. For reference the accepted normal values are also displayed in this table. The information in this table objectively shows that men from both groups have fragmented sleep, poor sleep efficiency, reduced TST and are awake after the onset of sleep for longer than expected. Despite remaining within the normal limits, there is a significant difference between the PLM of men with SRPE compared to men with SP (0.3 vs 13.5 per hour; *p* < 0.047).

**Table 2:** Sleep parameters measured on polysomnography in men with Stuttering priapism and Sleep-related painful erections.

Parameter	Accepted norms	Stuttering priapism (*n* = 12) Mean ± SD	SRPE (*n* = 8) Mean ± SD
Sleep latency	<30 min	10.8 ± 21.4	24.3 ± 41.5
Sleep efficiency	>85%	66.4 ± 24.2	61.0 ± 23.8
Total sleep time	420–560 min	327.8 ± 107.2	306.6 ± 121.8
Wake after sleep onset	<30 min	151.4 ± 107.7	175.3 ± 97.8
Arousal index	<10/h	8.7 ± 10.6	11.9 ± 9.3
Apnea hyponea index	<5/h	1.5 ± 1.45	2.95 ± 3.75
PLMS^a^	<15/h	0.3 ± 4.1	13.5 ± 20.2
REM awakenings (n)	0	0.9 ± 1.4	3.0 ± 1.4
Sleep fragmentation n (%)	0	8 (66.6)	7 (87.5)

Accepted norms derived from American Academy of Sleep Medicine Manual 2017 [10].

*PLMS* Periodic Limb Movements, *SRPE* Sleep related painful erections.

^a^*p* ≤ 0.05. *T*-Test.

Differences in the sleep architecture were also observed between the two groups. Men with SRPE tended to wake up during REM sleep very shortly after the onset of erection. Whereas, men with SP, would complete their REM cycle; then go back into deep sleep and then would wake with a painful erection much later than those with SRPE. Awakenings during REM sleep (that were experienced by men in the SRPE group) are considered abnormal (see Fig. 1) [8].

### Penile doppler ultrasound

**Fig. 2:**
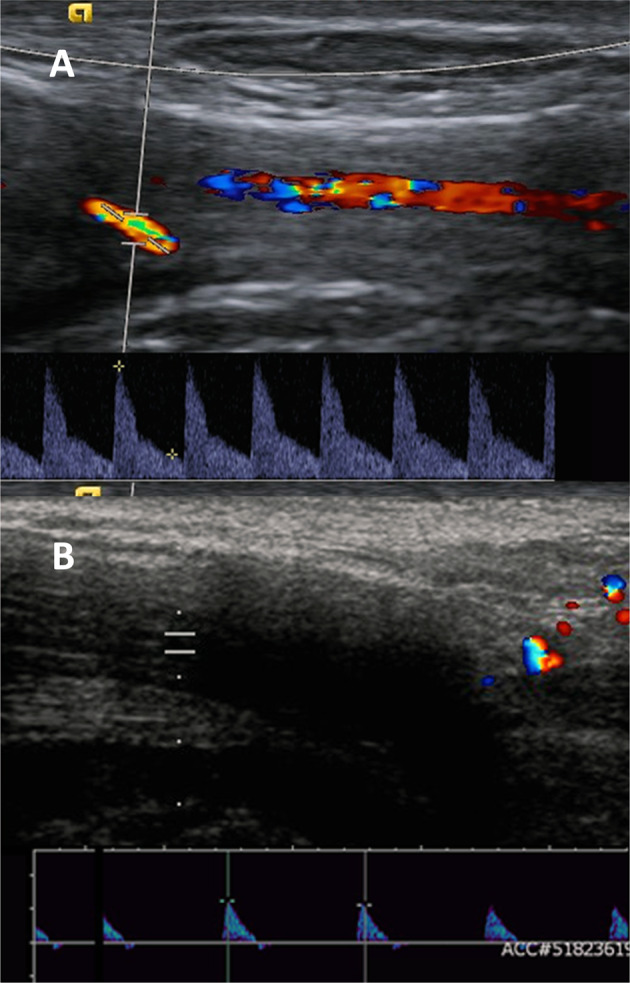
Unstimulated Penile Doppler Ultrasound findings in Groups 1 (Stuttering Priapism) and 2 (Sleep-related painful erections). **A** Group 1: Resting waveform in patients with stuttering priapism and multiple previous episodes of ischemia. This shows a high-flow low-resistance waveform. The peak systolic velocity is around 40 cm/s with the artery seen easily on color imaging and the forward flow in diastole make it a low resistance waveform. This is an abnormal resting waveform and is normally seen in phase two of the normal erectile cycle. **B** Group 2: Resting waveform in patients with SRPE and have not had an episode of ischemic priapism. This shows a low flow high resistance waveform. The peak systolic velocity is less than 10 cm/s, hence it is difficult to see the cavernosal artery on the color trace, and the lack of flow in diastole makes it a high resistance waveform. This is the resting waveform seen in normal men with a normal penis.

The median PSV in men with SP was significantly higher than that of men with SRPE (26.5 vs 15.5 cm/s; *p* = 0.03) (See Table 3). Men with SP typically had forward flow in end-diastole which is a low resistance waveform and suggests a reduced vascular tone which is an abnormal resting state. Despite this, no arterio-venous fistulae were identified. Men with SRPE showed zero or negative flow in end-diastole which is a high resistance waveform and is a normal resting waveform (see Fig. 2). There was a significant difference between the EDV in the two groups (2.5 vs −0.25 cm/s; *p* = 0.002).

**Table 3:** Median PSV and EDV on penile doppler ultrasound in men with stuttering priapism and sleep-related painful erections.

	Number of men (number of arteries)	PSV^a^	EDV^a^
Stuttering priapism	11 (22)	26.5 (22.5)	2.5 (4.5)
SRPE	7 (14)	15.5 (11.75)	−0.25 (3.375)

The PSV and EDV were significantly different in the groups with and without previous IP (*P* < 0.01; Mann–Whitney U Test).

*SRPE* Sleep related painful erections, *PSV* Peak systolic velocity, *EDV* End diastolic velocity.

^a^Median (Interquartile range) cm/s.

### Validated questionnaires

The PSQI is used to screen for sleep pathology. All men with bothersome nocturnal erections that were assessed for inclusion in this study had abnormal score of >5. The mean PSQI Global score for all participants in this study was 8.2 ± 1.7 suggesting poor sleep overall.

The IIEF is divided in domains of sexual function (erectile function, intercourse satisfaction, orgasm, desire, and overall satisfaction) over the previous 4 weeks [9]. Seven of the 20 men were excluded (SP group [*n* = 4]); SRPE group [*n* = 3]) due to lack of sexual activity in the preceding 4 weeks. The values of each domain are displayed in Table 4 and suggest a degree of sexual dysfunction.

**Table 4:** PSQI, IIEF, and SF36 questionnaire data in men with stuttering priapism and sleep-related painful erections.

Questionnaire	Domain	Stuttering priapism Mean ± SD	SRPE Mean ± SD	Total Mean ± SD	Accepted norms of healthy controls Mean ± SD
Pittsburgh Sleep Quality Index [10]	Global	7 ± 1.7	9.0 ± 1.7	8.2 ± 1.7	A score >5 indicates poor sleep
International Index of Erectile Function [11]	Erectile function	23.3 ± 10.6	19.4 ± 8.0	21.8 ± 9.5	25.8
	Orgasmic function	8.6 ± 2.3	9.4 ± 0.9	8.9 ± 1.9	9.8
	Sexual desire	8.1 ± 1.8	7.2 ± 2.4	7.8 ± 2.0	7.0
	Intercourse satisfaction	11.3 ± 4.1	7.6 ± 5.6	9.9 ± 4.9	10.6
	Overall satisfaction	8.1 ± 3.0	6.0 ± 2.1	7.3 ± 2.8	8.6
Short-Form 36—Health-related Quality of Life [16]	Physical functioning	71.3 ± 30.4	72.5 ± 35.5	71.8 ± 31.6	70.6 ± 27.4
	Limitation (physical)	43.8 ± 50.1	53.1 ± 50.8	47.5 ± 49.3	53.0 ± 40.8
	Limitation (Emotional)	27.8 ± 44.6	50 ± 53.5	36.7 ± 48.2	65.8 ± 40.7
	Energy/Fatigue	39.7 ± 25.8	44.4 ± 22.1	41.6 ± 23.9	52.2 ± 22.4
	Emotional wellbeing	48.0 ± 27.0	61.3 ± 24.7	53.3 ± 26.3	70.4 ± 22.0
	Social functioning	50.8 ± 33.0	55.3 ± 42.6	52.6 ± 36.1	78.8 ± 24.4
	Pain	63.3 ± 37.4	53.8 ± 33.3	59.5 ± 35.3	70.8 ± 25.5
	General health	43.4 ± 29.1	50.1 ± 33.3	46.1 ± 30.2	57.0 ± 21.1

*SRPE* Sleep related painful erections, *SD* Standard deviation.

The SF36 [10] assesses health-related QoL based on eight domains. The overall values and domains are displayed in Table 4. Each are scored from 0 to 100, with a lower score indicating a poorer health status. Overall, men in both groups scored poorly in most of the domains indicating a reduction in their QoL due to the poor health.

## Discussion

Often the primary complaint of men in both groups is poor sleep. Men with SP and SRPE were found to have fragmented sleep, poor sleep efficiency, reduced TST and increased wake after sleep onset time and REM sleep abnormalities [11]. *Ferini-Strambi et al*. described in his study of 18 patients with matched controls that the patients with SRPE had “reduced sleep efficiency increased wake after sleep onset, reduced REM and more fragmented REM.”[14]

This study provides further objective evidence of poor sleep patients with SRPE and SP. There is an increased prevalence of cardiovascular disease, malignancy, psychiatric illness, sepsis, and all-cause mortality in people that sleep poorly. Poor sleep is associated with traffic collisions, work-place accidents, and medical errors [1]. It is estimated that poor sleep costs the United Kingdom economy 1.86% gross domestic product or £35 Billion [2]. Referral to sleep specialists is warranted with the aim of helping to improve the sleep symptoms in these men and can aid in differentiating between these two conditions in equivocal cases.

Painful and bothersome nocturnal erections that result in poor sleep and daytime tiredness were experienced by all men in this study. Despite these similarities, it is unclear whether SP and SRPE are part of the same condition but with different severities; or two distinct conditions with different pathophysiology. It is also unclear whether one condition leads to the other i.e., Do men initially present with SRPE then progress to SP?

Based on the differences identified in this study, it is likely that SP and SRPE have a different underlying pathophysiology. There is increasing evidence in the literature to suggest this is the case. [6, 15, 20]

The pain experienced by the men with SP is ischemic in origin [3]. Men in this group have all had a previous corporal blood gas analysis that confirmed ischemia. They also have significantly longer nocturnal erections (60 min) and once awake took a significantly longer time to detumesce (25.7 min) when compared to men with SRPE. In men with SRPE the total duration was 18.5 min and the erection persisted after waking for 5.4 min. It would therefore be highly unlikely for an erection of 18.5 min to cause sufficient ischemia to result in pain that wakes up the patient. Especially given that these men do not experience painful erections during the daytime. It is therefore likely that pain experienced by men with SRPE is from a nonischemic cause. It was also noted that the symptoms of men with SP were worse in the second half of their nights’ sleep. It is postulated that this is due to rising testosterone levels in the early morning, however requires further investigation. Hypertonicity of the pelvic floor has also been cited as a possible cause of SRPE [5]. Future research using polysomnography and pelvic floor EMG could be considered.

Differences in the sleep architecture between the two groups was also identified. Sleep-related erections occurred during REM sleep in both groups. Normally, after each REM cycle, the individual reverts back to deeper sleep [4]. This was the case for men with SP, who were woken from deep sleep at the onset of penile pain. Whereas, the men with SRPE, would enter REM sleep, experience a sleep-related erection and be awoken from REM sleep very shortly afterwards. REM awakenings are considered abnormal.

Men with SRPE also had significantly higher PLM during sleep than men with SP. PLM are uncontrollable and repetitive limb movements that disrupt and occur during sleep. They are controlled by the medial preoptic area (MPOA) of the hypothalamus which is also responsible for the onset of sexual behaviors. Rats with lesions in the MPOA demonstrated reduced copulatory behavior [21, 22]. Central control of sleep related-erections are located in the lateral preoptic area, which is adjacent and connected to the MPOA [23]. This may offer a potentially exciting area of future basic science research in men with SRPE.

Sleep fragmentation can also be caused by sleep disordered breathing. Despite previous research suggesting a link between OSA and SP, this study was unable to find a clear association [24].

Differences in in the penile haemodynamics (PSV, EDV and resting waveforms) measured on PDU were identified between the two groups.

A normal high peripheral resistance waveform was observed in men in SRPE with absent or negative flow in diastole. This is the expected waveform in a healthy patient with a normal penis in the flaccid state and represents high vascular tone. An abnormal, low peripheral resistance waveform with forward flow in diastole was observed in the SP group. Typically, this waveform would be observed during the filling phase of erection or if an arterio-venous fistula is present; neither of these were observed in this study. A low peripheral resistance waveform implies a reduced vascular tone of the cavernosal smooth muscles. This is likely to represent pathologically relaxed sinusoidal smooth muscles.

These findings support a previous study that has too observed an elevated PSV and low resistance waveforms on PDU in men with SP and SCD [25]. The previous study explained these findings based on the molecular theory of SP. Animal model data identified that mice lacking endothelial and neuronal nitric oxide synthase (NOS) were more likely to experience priapic episodes. It is suggested that cavernosal smooth muscle that is deplete of NOS alters and downregulates various cellular signalling pathways. These include, cyclic guanosine mono-phosphate specific (cGMP), Phosphodiesterase-5A (PDE5A) and Rho A/Rho-kinase. Transfection of these mice with adenovirus encoded for endothelial NOS resulted in resolution of their priapism symptoms [26, 27].

It is argued, recurrent ischemia in men with SP causes destruction of the endothelium and a chronic reduction in endothelial NOS. This subsequently downregulates PDE5 and increases the activity of cGMP. This disrupts the haemodynamic penile equilibrium by reducing the sinusoidal peripheral resistance as demonstrated on PDU in this study. With an altered homeostatic resting state, the balance between erection and flaccidity is abnormal, making the point and stimulus at which erection is initiated and maintained different from that of a healthy individuals [25].

Based on the questionnaire data collected in this study, it shows that men in both groups not only sleep poorly, but experience a reduction in their health-related QoL and report sexual dysfunction. It is difficult to draw definite conclusions from this data as a comparative arm of healthy individuals was not included in the study design. However, when comparing the figures to the healthy controls in the Rosen et al. [9], IIEF study, the men in this study scored inferiorly in the domains of erectile function, orgasmic function, intercourse satisfaction, and overall satisfaction. When comparing the results in this study to the healthy controls in the Short-Form 36 study [10], the men scored worse in 7 out of the 8 domains, including physical limitation, emotional limitation, energy/fatigue, general health, pain, social functioning and emotional wellbeing. Given that 8 of these 20 men have SCD some of these findings may be expected. However, recognition and treatment of some of these factors may be required when treating these patients in clinic. The domains which scored the worst on the SF-36, were the psychological domain which may suggest that psychological support should be considered.

Men with SRPE were followed up for 47 ± 21 months. None of the men who were given an initial diagnosis of SRPE went on to subsequently experience episodes of IP. This suggests that men with SRPE do not progress to SP.

Distinguishing between men with SP and SRPE has important clinical application with regards to treatment (These differences are summarized in Table 5). This study has identified key differences between these groups to aid diagnosis and to prevent over-/under-treatment.

**Table 5:** Summary of the differences between men with SRPE and stuttering priapism.

Groups	SRPE	Stuttering priapism
Duration	<30 min	>30 min
Time to detumesce after waking	<10 min	>10 min
Awakenings during REM sleep	Yes	No
Timing within sleep	Spaced throughout the night	2nd half of the night
PDU waveform	High resistance	Low resistance

Multiple treatment options are available for SP, including PDE5i, α-agonists or androgen deprivation therapy (ADT). Treatment options for SP aim to prevent or suppress painful nocturnal erections [28]. Often, the most effective treatment for men with SP is ADT, which works by inhibition of physiological sleep-related erections [3, 28, 29]. ADT does however have significant associated side effects, and its use in men with SRPE could be considered over-treatment without careful counselling. In men with refractory symptoms, surgery (implantation of a penile prosthesis) can considered [28]. In men with SRPE, the primary aim is improvement in symptoms, therefore potential benefits of any treatment must be weighed up against the side effects. It would be equally inappropriate to under-treat a patient at risk of imminent IP and leave them risk of a prolonged episode with the associated morbidity.

The key limitation of this research is its small study population and lack of matched controls. Despite this, SP and SRPE are rare entities which are poorly understood and this study does provide some additional understanding. To further increase the number of participants in future studies, it is likely that collaborative research would be required. There was also a lack of control arm of healthy volunteers and or those with these conditions and good sleep, making comparison difficult. Access and compliance with NPT is overall scarce and poor and it is therefore of limited diagnostic utility in a clinical setting. Its role in this study was however helpful as it was objectively able to identify the onset of the painful erection prior the patient waking up and reporting it.

## Conclusion

SP and SRPE are rare entities with similar clinical features. This study has found multiple differences between these two conditions This is likely to represent differences in the underlying pathophysiology. Men with SRPE have associated sleep pathology (REM awakenings and PLM) on polysomnography and a normal PDU. This suggests a potential central (or sleep related) cause of their symptoms. Men with SP had an abnormal resting PDU with a low resistance waveform and no underlying sleep pathology identified. The men in this group have all had episodes of IP and recurrent episodes of prolonged erection. Each of these events causes ischemia and damages the sinusoidal endothelium and cavernosal smooth muscles thus altering the haemodynamics of the penis. This is a hypothesis-generating observational study.
